# The efficacy and safety of Iodine-131-metaiodobenzylguanidine therapy in patients with neuroblastoma: a meta-analysis

**DOI:** 10.1186/s12885-022-09329-2

**Published:** 2022-02-28

**Authors:** Huihui He, Qiaoling Xu, Chunjing Yu

**Affiliations:** Department of Nuclear Medicine, Affiliated Hospital of Jiangnan University, Wuxi, China

**Keywords:** ^131^I-MIBG, Neuroblastoma, Neuroendocrine tumor, Clinical trials, Meta-analysis

## Abstract

**Objective:**

Neuroblastoma is a common extracranial solid tumor of childhood. Recently, multiple treatments have been practiced including Iodine-131-metaiodobenzylguanidine radiation (^131^I-MIBG) therapy. However, the outcomes of efficacy and safety vary greatly among different studies. The aim of this meta-analysis is to evaluate the efficacy and safety of ^131^I-MIBG in the treatment of neuroblastoma and to provide evidence and hints for clinical decision-making.

**Methods:**

Medline, EMBASE database and the Cochrane Library were searched for relevant studies. Eligible studies utilizing ^131^I-MIBG in the treatment of neuroblastoma were included. The pooled outcomes (response rates, adverse events rates, survival rates) were calculated using either a random-effects model or a fixed-effects model considering of the heterogeneity.

**Results:**

A total of 26 clinical trials including 883 patients were analyzed. The pooled rates of objective response, stable disease, progressive disease, and minor response of ^131^I-MIBG monotherapy were 39%, 31%, 22% and 15%, respectively. The pooled objective response rate of ^131^I-MIBG in combination with other therapies was 28%. The pooled 1-year survival and 5-year survival rates were 64% and 32%. The pooled occurrence rates of thrombocytopenia and neutropenia in MIBG monotherapy studies were 53% and 58%. In the studies of ^131^I-MIBG combined with other therapies, the pooled occurrence rates of thrombocytopenia and neutropenia were 79% and 78%.

**Conclusion:**

^131^I-MIBG treatment alone or in combination of other therapies is effective on clinical outcomes in the treatment of neuroblastoma, individualized ^131^I-MIBG is recommended on a clinical basis.

## Introduction

Neuroblastoma is a common extracranial solid tumor of childhood, accounting for approximately 8% of total pediatric malignant tumors [1, 2]. It derives from primitive sympathetic nervous system tissue and arises mostly from adrenal medulla or paraspinal ganglia of the neck, chest, abdomen, or pelvis [3]. Statistically, neuroblastoma occurs more common in boys than in girls, however, the potential causes remain long-standing mysteries [4]. Furthermore, over one-third of the patients are diagnosed at the age of < 12 months and the median age at diagnosis is 17 months More than 50% of children present with widely metastatic disease [5].

The type of therapy for neuroblastoma depends on risk group in which a patient identifies [5, 6]. Risk stratification is determined according to a patient’s International Neuroblastoma Risk Group (INRG) stage, age, histological condition of tumor, degree of tumor differentiation, and *et al* *[*6*]*. Typically, in low-risk patients may be monitored for spontaneous differentiation or regression of tumor and either chemotherapy or radiation may not be necessary in these patients. Conversely, chemotherapy may be used in patients with intermediate or high risk. Moreover, patients with high risk may receive stem cell transplant, immunotherapy and surgery.

Despite multiple choices of treatment mentioned above, patients with neuroblastoma continue to be at high risk of treatment failure [7–10]. Unfortunately, patients with refractory or relapsed neuroblastoma suffer from poor prognosis, while novel therapy is in need [11]. Currently, there is no consensus on the optimal treatment for neuroblastoma.

Meta-iodobenzylguanidine (MIBG) is an analogue of adrenergic neuron blockers, it shows high affinity to cells of the sympathetic nervous system and by neoplasms arised from them, such as neuroblastoma [9]. Interestingly, Iodine-131 labeled MIBG (^131^I-MIBG) was used to treat neuroendocrine tumors including neuroblastoma after the development of MIBG [12, 13]. Since then, findings on the treatment role of ^131^I-MIBG have occurred [14, 15]. The first I-131 MIBG therapy for neuroblastoma were reported in 1986 [16]. In the following years, several other groups also conducted phase I or phase II clinical trials on the efficacy and safety of ^131^I-MIBG on the treatment of neuroblastoma. However, the objective response (partial or complete response) rate varied widely, from 30% to 71% [14, 15, 17–24].

As far as we are concerned, a few studies limited to small sample sizes and heterogeneity of treatment outcomes have investigated the efficacy of ^131^I-MIBG for the treatment of neuroblastoma. The aim of this study was to conduct a meta-analysis by collating the available evidence to generate an accurate and sounding assessment of the efficacy and safety of ^131^I-MIBG monotherapy and ^131^I-MIBG in combination with other agents, and subsequently to provide evidence and hints for clinical implement and decision-making.

## Materials and Methods

### Statement

This meta-analysis was entirely based on previous published studies which had declared ethical approvals, and no original clinical raw data of the published results were collected or utilized, thereby ethical approval was not conducted for this study. This review was conducted on the basis of the Preferred Reporting Items for Systematic Reviews and Meta-analysis (PRISMA) [25].

### Literature search and selection criteria

We conducted a comprehensive literature search of online databases of the Medline (via PubMed), Embase database and the Cochrane Library from inception to May 31, 2021. Our search strategy was (("Iodine Radioisotopes"[Mesh] OR ("iodine radioisotopes"[MeSH Terms] OR ("iodine"[All Fields] AND "radioisotopes"[All Fields]) OR "iodine radioisotopes"[All Fields] OR "therapy"[All Fields]) AND " neuroblastoma "[All Fields]. Additionally, we manually searched the reference lists of all accepted papers to ensure that no studies were missed. All articles were published in English. Studies that met the following criteria were enrolled for this meta-analysis: (1) clinical trials designed to evaluate the efficacy of ^131^I-MIBG or ^131^I-MIBG in combination with other therapies (radiation sensitizer, myeloablative chemotherapy, etc.) in untreated, relapsed or refractory neuroblastoma; (2) data available for the extraction or calculation tumor treatment response rates, survival and adverse events. Once studies recruited participants over the same period or from the same study centers, only the study with the largest sample size or yielding the most pertinent outcomes was included to avoid duplications. All the potentially relevant papers were reviewed independently by two investigators (HH and QX) and disagreement were resolved by discussion and a third reviewer (CY) was involved in case that no consensus was achieved.

### Data extraction and quality assessments

Two independent reviewers screened the titles and abstracts of articles to judge whether they meet the inclusion criteria. Thereafter a full-text reading of the literature was performed for the final inclusion. Details on patients’ characteristics, ^131^I-MIBG dose and schedule, tumor response rates were also extracted independently by two investigators. The main clinical endpoints were tumor response rate, including complete response (CR), partial response (PR), progressive disease (PD), stable disease (SD), minor response (MR), survival rates, and adverse events (AEs) rates. Objective response was defined as patients either undergo a partial or complete response. Event-free survival (EFS) rates and overall survival (OS) rates in each study was also extracted. We used the Newcastle-Ottawa Quality Assessment Scale to assess the methodological quality of enrolled studies [26]. The Newcastle-Ottawa Quality Assessment Scale contains 3 categories (quality selection, comparability and outcome) across which cohort studies are assessed for quality.

### Statistical analysis

All statistical analyses were conducted using R 4.1.2 software package. The efficacy and safety of ^131^I-MIBG treatment in neuroblastoma was assessed depending on the indicators aforementioned. A Cochran Q test was used to assess heterogeneity between studies and I^2^ statistic was used to investigate the magnitude of the heterogeneity. Pooled rates of objective response, SD, PD, MR, 1-year survival, 5-year survival, AEs and their respective 95% confidence intervals (CIs) were calculated with a random-effects model or a fixed-effects model. If I^2^ value was >50%, a random-effects model was used, otherwise we used a fixed-effects model [27]. A sensitivity analysis was conducted in order to check the stability of pooled outcomes. Furthermore, an Egger’s test was performed to assess the potential publication bias. A two-tailed P value <0.05 was regarded as statistically significant.

## Results

### Identification of relevant studies

**Fig. 1:**
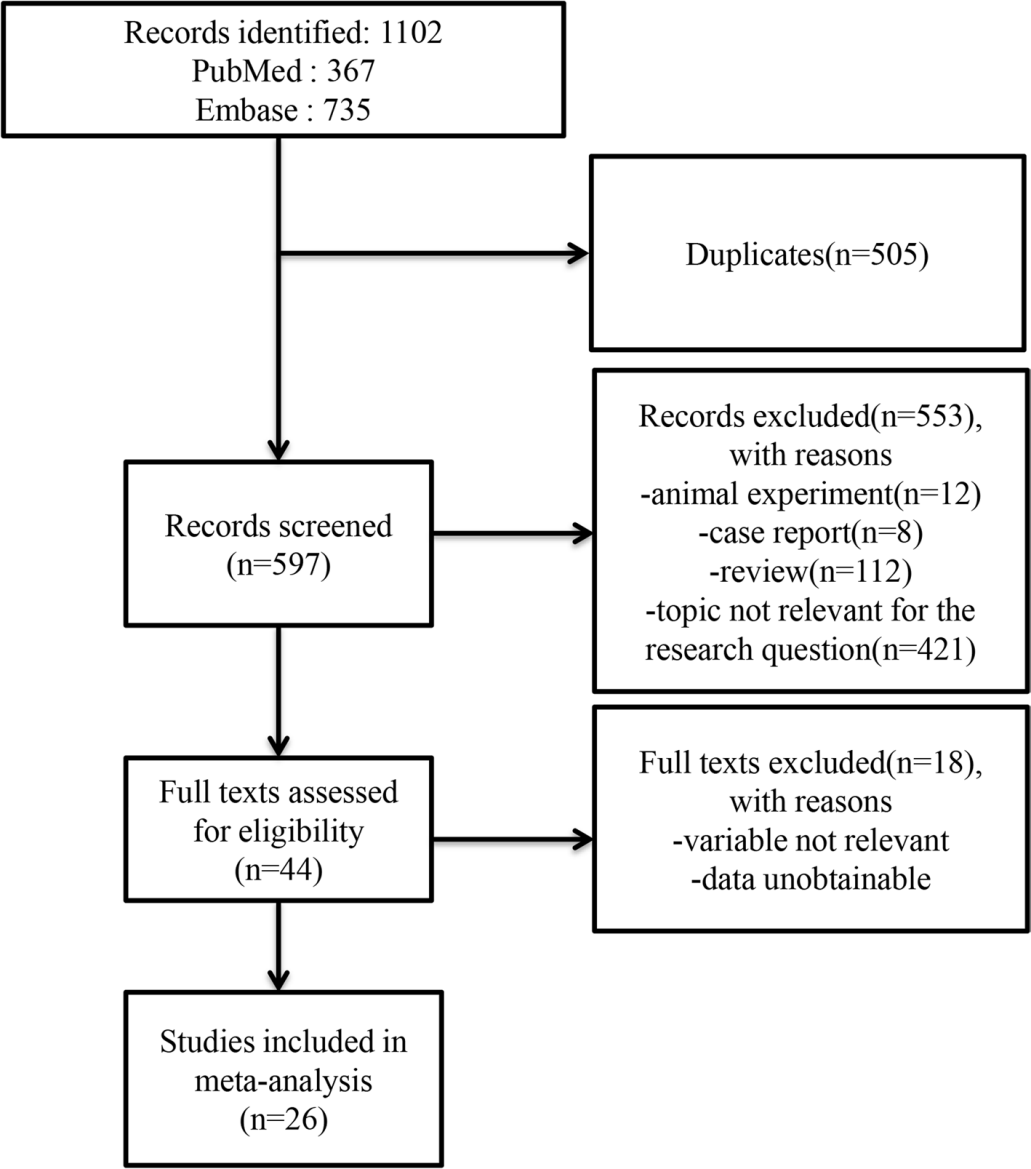
Flow diagram of study selection process

A total of 1102 articles were identified from the process of database search. A total of 26 articles were identified for analysis. Figure 1 shows the details of the literature search and study selection process. The enrolled 26 studies containing a total of 883 patients with diagnosed neuroblastoma, provided relevant outcomes that met the inclusion criteria in this meta-analysis. The majority of these studies did not have a control group. These clinical trials were conducted in UK, USA, Italy, Thailand, Japan and Netherlands. All studies included demonstrated low risk of bias. More details of the studies included was shown in Table 1.

**Table 1:** Characteristics of the studies included in the meta-analysis

Year	Name of First Author	Country	Trial design	Schedule	Response criteria	Patients Enrolled	Tumor response			
							CR/PR	SD	PD	MR
1991	Hutchinson [28]	NS^a^	Single-arm Phase I	Doses ranged from 50-220 mCi, with cumulative doses of 50-654 mCi in one to three doses		14	4	-	-	2
1991	Klingebiel [29]	Germany	NS	NS	NS	47	9	-	-	-
1991	Matthay [30]	NS	Single-arm Phase I	100-400 mCi/m^2^/course		11	2	2	7	0
1991	Troncone [31]	Italy	Single-arm Phase I	single doses (2.6-9.5 GBq)		11^a^	2	4	2	1
1992	Lashford [24]	UK	Single-arm Phase I	NS	ENSG Criteri a[24]	25	8	9	7	-
1994	Hoefnagel [14]	Netherlands	Single-arm Phase II	First 200mCi, If necessary, more cycles with100mCi at 4 weeks intervals	NS	31	22	8	-	-
1995	de Kraker [15]	Netherlands	Single-arm Phase II	First 200mCi, If necessary, more cycles with100mCi at 4-6 weeks intervals	INR C[32]	33	19	11	3	-
1999	Garaventa [17]	Italy	Single-arm Phase II	67.5-148mCi 1-5 courses	INRC	43	13	-	5	25
2005	Howard [18]	USA	Single-arm Phase II	3-19mCi/kg 2 to 4 courses	INRC	28	11	8	8	1
2007	Matthay [19]	USA	Single-arm Phase II	12 or 18mCi/kg	INRC	164	59	55	44	5
2008	de Kraker [23]	Netherlands	Single-arm Phase II	200 mCi for the first infusion and 100–150 mCi for the second and all subsequent infusions.	INRC	41^b^	27	5	4	4
2009	Matthay [20]	USA	Single-arm Phase I	Day 0 and day 14, 12-21mCi/kg	RECIS T[33]	20	10	3	7	8
2011	Johnson [21]	USA	Single-arm Phase II	18mCi/kg If necessary, additional 18mCi/kg were received within 100 days.	INRC	117	35	52	30	-
2011	Mastrangelo [34]	Italy	Pilot study	^131^I-MIBG combined with other therapies	INRC	13	6	-	-	1
2011	Polishchuk [22]	USA	Single-arm Phase II	17.8 millicuries (mCi)/kg	INRC	39	18	17	2	2
2012	DuBois [35]	USA	Single-arm Phase I	^131^I-MIBG combined with other therapies	NANT Response Criteri a[35]	24	6	-	-	-
2013	Kushner [36]	USA	NS	^131^I-MIBG combined with other therapies	INRC	3	1	2	0	0
2015	DuBois [37]	USA	Single-arm Phase I, II	^131^I-MIBG combined with other therapies	NANT Response Criteria	32	9	-	-	-
2015	DuBois [38]	USA	Single-arm Phase I	^131^I-MIBG combined with other therapies	NANT Response Criteria	27	7	-	-	-
2015	Kraal [39]	Netherlands	Single-arm Phase II	^131^I-MIBG combined with other therapies	INRC	16	9	-	-	-
2015	Yanik [40]	USA	Single-arm Phase II	^131^I-MIBG combined with other therapies	INRC	49	7	26	6	10
2016	George [41]	UK	NS	^131^I-MIBG monotherapy	INRC	25	15	8	-	-
2016	Modak [42]	USA	Single-arm Phase II	^131^I-MIBG combined with other therapies	INRC	19	0	-	7	-
2019	Genoll a[43]	Spain	NS	^131^I-MIBG combined with other therapies	INRC, RECIST	10	7	2	1	0
2020	Anongpornjossakul [44]	Thailand	NS	mean dose of 136 mCi per treatment	RECIST 1.1 [45]	22	7	3	12	0
2020	Kayano [46]	Japan	NS	single dose of 444 to 666 MBq/kg	RECIST 1.1	19^b^	5	10	3	0

a: 2 patients were not evaluable. b: 1 patient was not evaluable. NS: Not specified. RECIST, Response Evaluation Criteria in Solid Tumors.

*INRC* the International Neuroblastoma Response Criteria. *NANT* the New Approaches to Neuroblastoma Therapy. *ENSG* European Neuroblastoma Study Group

### Efficacy of ^131^I-MIBG monotherapy

**Fig. 2:**
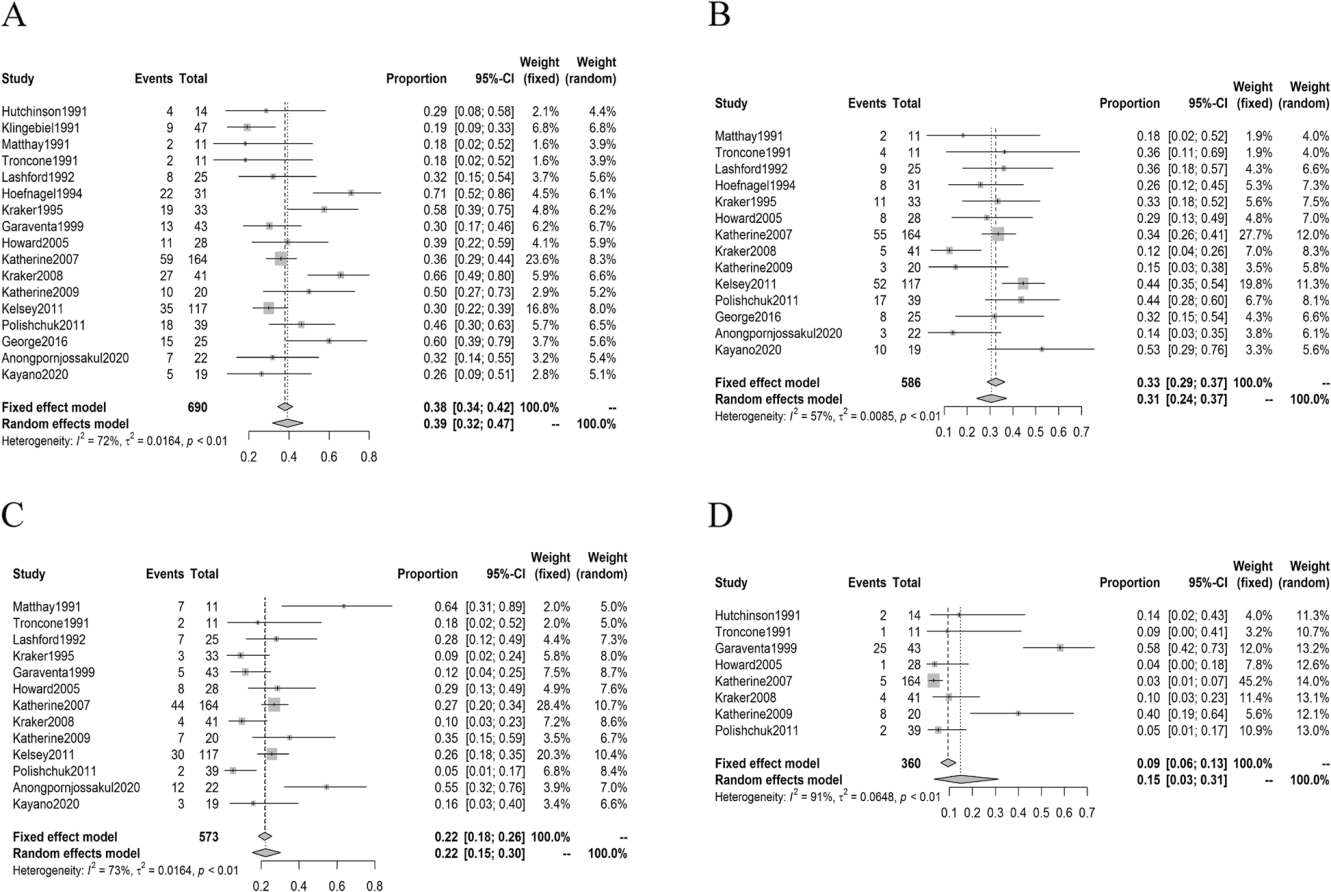
Forest plots of response rates in studies of ^131^I-MIBG monotherapy. **A**: Forest plot of objective response rates in studies of ^131^I-MIBG monotherapy. **B**: Forest plot of SD rates in studies of ^131^I-MIBG monotherapy. **C**: Forest plot of PD rates in studies of ^131^I-MIBG monotherapy. D: Forest plot of MR rates in studies of ^131^I-MIBG monotherapy

The numbers of articles included in the evaluation of rates of objective response, SD, PD and MR were 17, 14, 13 and 8, respectively. The objective response rates ranged from 30% to 71%. The pooled objective response rate was 39% (95% CI: 32%-47%) as calculated utilizing the random-effects model. The pooled rates of SD, PD and MR were 31% (95% CI: 24%, 37%), 22% (95% CI: 15%, 30%) and 15% (95% CI: 3%, 31%), respectively (Fig. 2).

### Efficacy of ^131^I-MIBG combined with other therapies

**Fig. 3:**
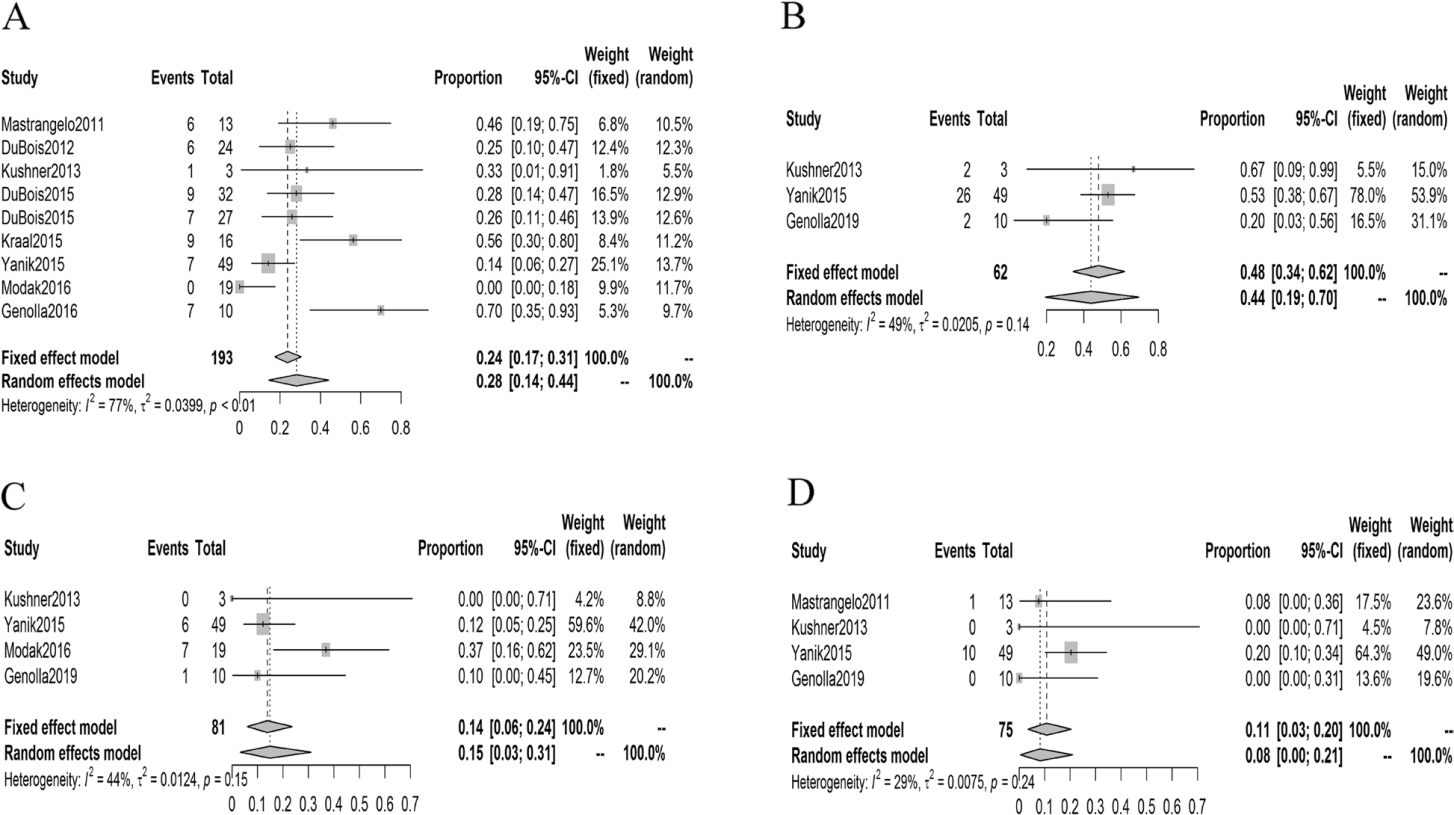
Forest plots of response rates in studies of ^131^I-MIBG combined with other therapies. **A**: Forest plot of objective response rates in studies of ^131^I-MIBG combined with other therapies. **B**: Forest plot of SD rates in studies of ^131^I-MIBG combined with other therapies. **C**: Forest plot of PD rates in studies of ^131^I-MIBG combined with other therapies. **D**: Forest plot of MR rates in studies of ^131^I-MIBG combined with other therapies.

Nine studies investigating the efficacy and safety of ^131^I-MIBG in combination with other therapies were included. Two studies reported the combination of ^131^I-MIBG and radiation sensitizer, 7 studies reported the combined employment of ^131^I-MIBG with chemotherapeutic agents namely cisplatin, cyclophosphamide, etoposide, vincristine, doxorubicin, irinotecan, and topotecan. The pooled objective response rate of ^131^I-MIBG in combination with other therapies was 28% (95% CI: 14%, 44%). The pooled rates of SD, PD and MR were 48% (95% CI: 34%, 62%), 14% (95% CI: 6%, 24%) and 11% (95% CI: 3%, 20%), respectively (Fig. 3). The pooled objective response rate of ^131^I-MIBG in combination with chemotherapy was 35% (95% CI: 20%, 52%).

### Survival

**Fig. 4:**
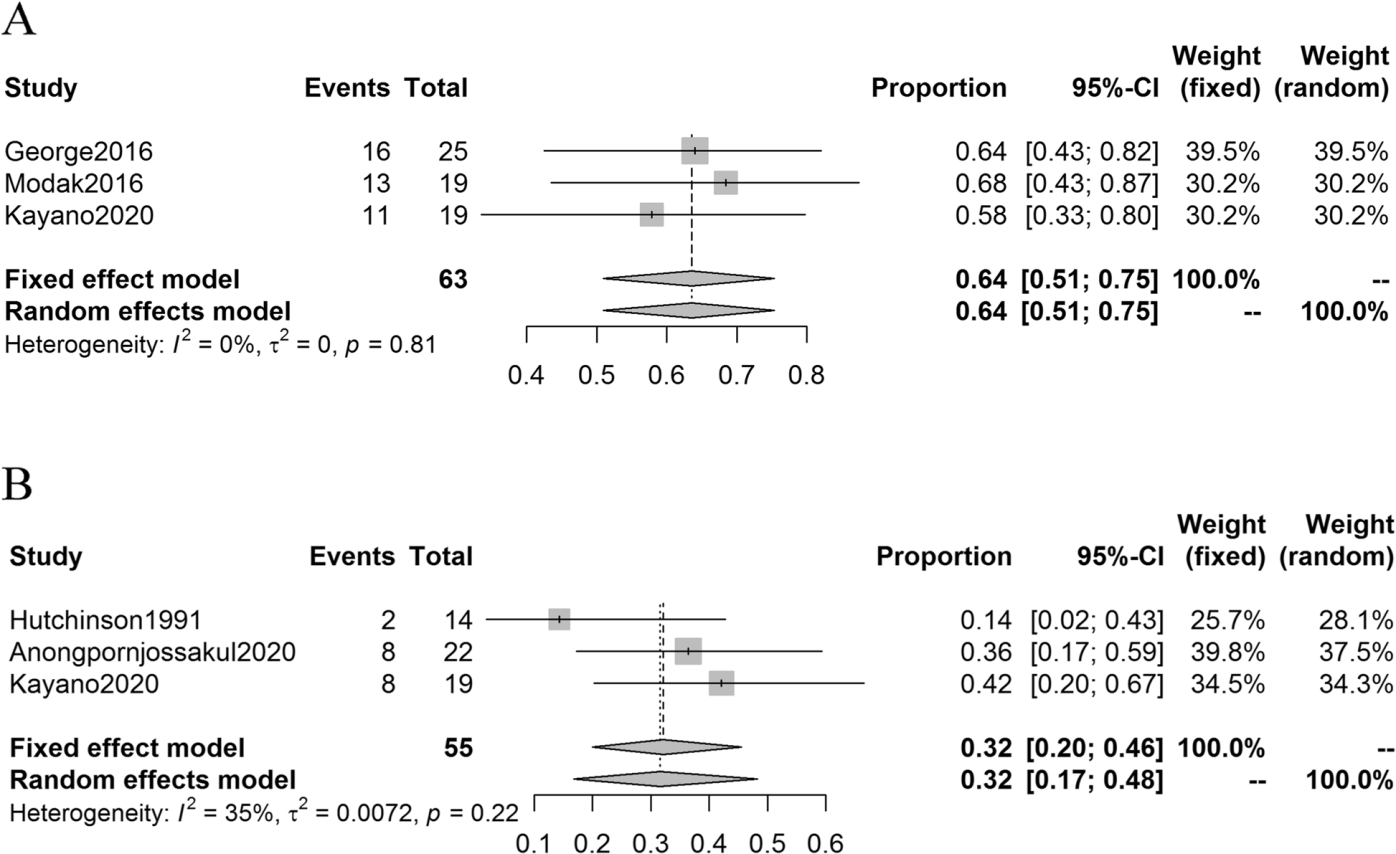
Forest plots of 1-year and 5-year survival rates in studies included. **A**: Forest plot of 1-year survival rates in studies included. **B**: Forest plots of 5-year survival rates in studies included

The pooled 1-year survival and 5-year survival rates were 64% (95% CI: 51%, 75%) and 32% (95% CI: 20%, 46%) (Fig. 4). Three studies reported median event-free survival which ranged from 10 to 16 months.

### Toxicity

**Fig. 5:**
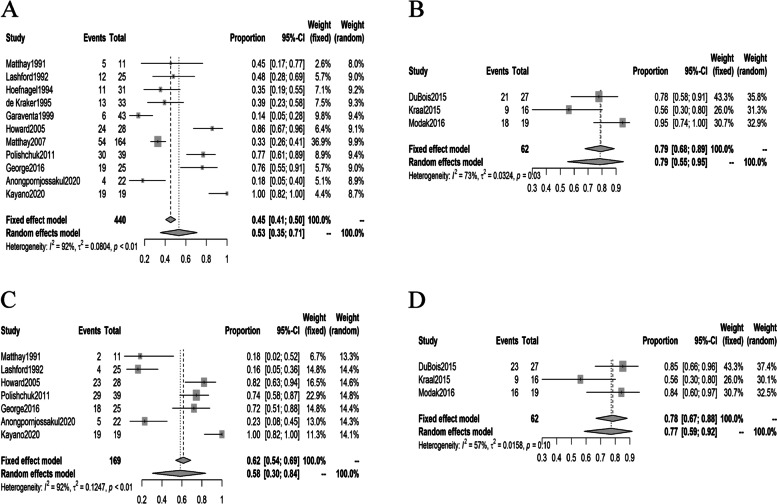
Forest plots of AEs rates in studies included. **A**: Forest plot of thrombocytopenia occurrence rates in MIBG monotherapy. **B**: Forest plot of thrombocytopenia occurrence rates in studies of ^131^I-MIBG combined with other therapies. **C**: Forest plot of neutropenia occurrence rates in MIBG monotherapy. **D**: Forest plot of neutropenia occurrence rates in studies of ^131^I-MIBG combined with other therapies

With regard to AEs rates, the major toxicity reported by studies included was hematologic, thrombocytopenia and neutropenia were the most frequently reported. The pooled occurrence rates of thrombocytopenia and neutropenia in MIBG monotherapy studies were 53% (95% CI: 35%, 71%) and 58% (95% CI: 30%, 84%), respectively. As in the studies of ^131^I-MIBG combined with other therapies, the pooled occurrence rates of thrombocytopenia and neutropenia were 79% (95% CI: 55%, 95%) and 78% (95% CI: 67%, 88%), respectively (Fig. 5).

### Heterogeneity and publication bias

The results of the heterogeneity tests in rates of objective response, SD, PD, MR, and occurrence rates of thrombocytopenia and neutropenia in studies of ^131^I-MIBG monotherapy were as follows: I^2^ = 72% (*p* < 0.01), I^2^ = 57% (*p* < 0.01), I^2^ = 73% (*p* < 0.01) I^2^ = 91% (*p* < 0.01), I^2^ = 92% (*p* < 0.01) and I^2^ = 92% (*p* < 0.01) (see Figs. 2 and 5). In the pooled analysis of studies of ^131^I-MIBG combined with other therapies, the I^2^ values of objective response, SD, PD, MR, and occurrence rates of thrombocytopenia and neutropenia were 77% (p < 0.01), 49% (*p =* 0.14), 44% (*p =* 0.15), 29% (*p =* 0.24), 73% (*p =* 0.03) and 57% (*p =* 0.10) (see Figs. 3, 5). Egger’s tests for publication bias yielded *p* values of 0.614, 0.240, 0.834, 0.243, 0.1761 and 0.5356 for rates of objective response, SD, PD, MR and occurrence rates of thrombocytopenia and neutropenia in studies of ^131^I-MIBG monotherapy. In the pooled analysis of studies of ^131^I-MIBG combined with other therapies, the corresponding p values objective response, SD, PD, MR and occurrence rates of thrombocytopenia and neutropenia were, 0.210, 0.7808, 0.9663, 0.1823, 0.8347, 0.4111, respectively.

### Sensitivity Analysis

We performed the sensitivity analysis to assess the impacts of each single study on the pooled outcomes. For the analysis of MR in studies of ^131^I-MIBG monotherapy, the sensitivity analysis revealed that result from Garaventa’s study may have impacts on the outcomes, suggesting that the study was probably to be the main source of heterogeneity. Nevertheless, after excluding single study one after another, the pooled rates demonstrated the robustness of the results.

## Discussion

Neuroblastoma is the most common extracranial solid tumor in children, and is regarded as the most common malignant tumor in infants so far [47]. Treatment outcomes vary significantly among patients with neuroblastoma, as patients with low risk of neuroblastoma fare well with little or no treatment, whereas high-risk children was diagnosed with metastatic disease or have an event-free survival (EFS) of approximately 50% despite multimodality therapeutic schedule that give rise to significant long-term side-effects [48–50]. Iodine-131-metaiodobenzylguanidine (^131^I-MIBG) has been used to treat neuroblastoma with a rapid development in recent decades. The efficacy and safety of ^131^I-MIBG therapy remains the most concerned issues. However, the outcomes varied greatly in different investigations. A meta-analysis was conducted by pooling cumulative evidence from institutional reports and some early phase trials to make a more comprehensive evaluation of the efficacy and safety of ^131^I-MIBG therapy in patients with neuroblastoma. The pooled objective response rate in patients treated with ^131^I-MIBG monotherapy and ^131^I-MIBG in combination with other therapies were 39% and 28%. The reason for this unexpected difference may be that patients in some studies were heavily prior-treated with other therapies. Furthermore, dose heterogeneity among studies could affect the outcomes of therapies and may partially explain the variation in responses. Unfortunately, the schedules varied on study level and we have no access to doses on patient level, in light of this, subgroup analysis based on doses in each study was not performed. With respect to adverse events, thrombocytopenia and neutropenia were the most frequently reported in the majority of investigations enrolled. As found in this study, the pooled occurrence rates of adverse events of ^131^I-MIBG combined with other therapies were higher than that of ^131^I-MIBG alone. Because most patients who receive ^131^I MIBG with other therapies have been treated with several other intensive therapies before and the adverse events of the combined therapy tended to be more common. Furthermore, the pooled 1-year survival and 5-year survival rates in this study were 64% and 32%.

In this meta-analysis, we did a detailed literature search in Medline, Embase and the Cochrane Library databases to enhance the probability of retrieving all relevant studies as we can. Data extraction was conducted by two independent investigators using a well-designed form. Moreover, the heterogeneity in the studies included was assessed. The results of the meta-analysis showed that there were significant heterogeneities in the majority of indicators. The potential reasons may be attributed to differences in inclusion criteria of the study participants, study design, drug compliance, median lines of prior therapy in each study, batch of drug and other relevant factors. Furthermore, Egger’s tests for publication indicated that no potential publication bias was observed in the studies included. Despite the existences of heterogeneity, the results of this analysis may provide hints and assistances for a profile of clinical trials detecting the efficacy and safety of ^131^I-MIBG therapy with larger sample sizes and longer follow-ups.

Our study has provided a comprehensive evaluation of the efficacy and safety of ^131^I-MIBG in the treatment of neuroblastoma. Currently, the best available evidence on the efficacy is derived from several single-arm phase II clinical trials. The findings of this meta-analysis suggest that ^131^I-MIBG is an effective agent with acceptable toxicity in patients with neuroblastoma. Due to the heterogeneity of patients’ characteristics and low number of relapsed and refractory neuroblastoma, large sample-sized randomized controlled trials are difficult to be performed, nevertheless, individualized ^131^I-MIBG therapy alone or in combination with other agents is recommended in clinical setting.

## Data Availability

The datasets used and/or analysed during the current study available from the corresponding author on reasonable request.
